# Protein microarray: sensitive and effective immunodetection for drug residues

**DOI:** 10.1186/1472-6750-10-12

**Published:** 2010-02-16

**Authors:** Li Zhong, Wei Zhang, Cindy Zer, Kun Ge, Xu Gao, Kemp H Kernstine

**Affiliations:** 1 Hebei University College of Life Sciences, Baoding 071002, China; 2 Division of Radiation Biology, Department of Cancer Biology, City of Hope and Beckman Research Institute, 1500 Duarte Road, Duarte, CA 91010, USA; 3 Lung Cancer and Thoracic Oncology Program, City of Hope and Beckman Research Institute, 1500 Duarte Road, Duarte, CA 91010, USA; 4 Michigan State University College of Natural Science, East Lansing, MI 48824, USA

## Abstract

**Background:**

Veterinary drugs such as clenbuterol (CL) and sulfamethazine (SM_2_) are low molecular weight (<1000 Da) compounds, or haptens, that are difficult to develop immunoassays due to their low immunogenicity. In this study, we conjugated the drugs to ovalbumin to increase their immunogenicity for antiserum production in rabbits and developed a protein microarray immunoassay for detection of clenbuterol and sulfamethazine. The sensitivity of this approach was then compared to traditional ELISA technique.

**Results:**

The artificial antigens were spotted on microarray slides. Standard concentrations of the compounds were added to compete with the spotted antigens for binding to the antisera to determine the IC_50_. Our microarray assay showed the IC_50 _were 39.6 ng/ml for CL and 48.8 ng/ml for SM_2_, while the traditional competitive indirect-ELISA (ci-ELISA) showed the IC_50 _were 190.7 ng/ml for CL and 156.7 ng/ml for SM_2_. We further validated the two methods with CL fortified chicken muscle tissues, and the protein microarray assay showed 90% recovery while the ci-ELISA had 76% recovery rate. When tested with CL-fed chicken muscle tissues, the protein microarray assay had higher sensitivity (0.9 ng/g) than the ci-ELISA (0.1 ng/g) for detection of CL residues.

**Conclusions:**

The protein microarrays showed 4.5 and 3.5 times lower IC_50 _than the ci-ELISA detection for CL and SM_2_, respectively, suggesting that immunodetection of small molecules with protein microarray is a better approach than the traditional ELISA technique.

## Background

Veterinary drugs are widely used in modern agricultural practice for therapeutic and prophylactic purposes. Unfortunately, illegal and abusive usage of veterinary drugs can cause long-term and short-term public health hazard [1]. Monitoring of edible animal products for the presence of veterinary drug residues is an essential process in ensuring the safety of the food supply. The complexity of the biological matrices and the huge variety of drug residues preclude the use of a single analytical method. Currently, liquid chromatography (LC) combined with tandem mass spectrometric detection is the preferred technique in a large majority of all cases for quantification and confirmation [2-4]. Other chromatographic methods such as high performance liquid chromatography (HPLC) can be used for multiple residues screening, but the need for extensive sample pretreatment, specialized equipment and highly trained personnel make most chromatographic methods poorly suited for screening purposes. Microbial survival assays and immunoassays are more suitable for screening. However, microbiological assays are time consuming, and the identity of the offending compound is not immediately known. On the other hand, immunochemical detections such as ELISA have a fast turnaround, there is no need for any extensive sample preparation, and the compound is immediately identified. ELISAs are, therefore, one of the most commonly employed screening methods.

Protein microarray as an emerging technology has many advantages over the traditional ELISA method. It is a versatile, miniature and high-throughput platform that can be adapted for a variety of screening uses. Protein microarrays are already widely used to perform high though-put drug screens, to study protein-protein interactions, and have great potential for disease diagnosis [5,6]. The technology can also be used to detect veterinary drug residues. Here, we describe our protein microarray immunodetection assay [7,8] for two commonly abused veterinary drugs: clenbuterol (CL) [9] and sulfamethazine (SM_2_) [10]. We compared its sensitivity to traditional ELISA-based method, and tested the applicability of the assay to complex biological material.

## Methods

### Reagents and chemicals

The following reagents were purchased from Sigma-Aldrich, St. Louis, MO, USA: clenbuterol (CL), sulfamethazine (SM_2_), bovine serum albumin (BSA), ovalbumin (OVA), Freund's adjuvant complete (FCA), Freund's adjuvant Incomplete (FIA), Tween-20 and N', N-Dimethylformamide (DMF). Cy3-goat-anti-rabbit IgG, and HRP-goat-anti-rabbit IgG were purchased from Jackson ImmunoResearch, West Grove, PA, USA. 3,3'5,5'-Tetramethyl Benzidine dihydrochloride (TMB) was purchased from Amresco, Inc., Solon, OH, USA.

### Hapten-carrier conjugation

The aromatic primary amine on CL and SM_2 _were activated by diazotization and coupled to the phenol group of tyrosine residues on ovalbumin (OVA) as a carrier protein [11]. The detailed conjugation procedures are as follows: 10 mg of each hapten were first acidified (CL with 400 μl of 1 M HCl; SM_2 _with 250 μl 0.5 M H_2_SO_4_). Then, 2% NaNO_2 _was slowly added with stirring. After 1 h, 1 ml of 0.1 M carbonate buffer solution (pH 9.6) containing 35 mg OVA was added. The pH was maintained between 9 and 9.6 through the periodic addition of 0.5 M NaOH. The solution was allowed to stir overnight at 4°C. The conjugated product was dialyzed into PBS for 72 h, with PBS replacement at 8 h intervals. The synthetic products were combined with the same volume of sterile 80% glycerol and then frozen at -20°C. The formation of the artificial antigens was monitored by UV absorbance from 200 to 400 nm (TU-1810 V-spectrophotometer, Beijing Purkinje General Instruments, Beijing, China). Within this wavelength range, the haptens, OVA and the respective conjugates have distinct absorbance patterns and thus could be used to identify the formation of the conjugated product.

The efficiency of the diazotization was determined by the coupling ratios of the haptens to OVA. The conjugation ratios of CL to OVA and SM_2 _to OVA are 16:1 and 3:1, respectively.

### Antibody Production

One mg of each immunogen (CL-OVA and SM_2_-OVA) were diluted with sterile saline and combined with an equal volume of complete Freund's adjuvant for the initial subcutaneous injection into New Zealand White Rabbits (SLAC Laboratory Animals, Shanghai, China). Second and third injections were mixed with incomplete Freund's adjuvant, and no adjuvant was used for the forth. The injections were 15 days apart, and two rabbits were immunized with each immunogen. Blood was collected before the start of immunizations as a negative control, and 10 days after each immunization for titer monitoring. 2 ml of blood was collected each time from the central ear artery and allowed to clot and retract at 37°C for 2 h and overnight at 4°C. The blood samples were then centrifuged at room temperature for 15 minutes at 3000 rpm and the sera were decanted into sterile tubes.

### Antibody Titer Monitoring

Titers of the polyclonal antibodies against the two immunogens were determined by indirect ELISA. 96 well plates were coated with CL or SM_2 _artificial antigens overnight at 4°C. The plates were washed 3 times with PBST (0.5%Tween-20 in PBS), and blocked with 150 μl of 0.1% (w/v) OVA per well at 37°C for 1.5 h. Antisera from each injection were diluted from 1:200 to 1:25,600 on a two-fold dilution series and 100 μl were applied to each well. Undiluted pre-immune sera were also assayed as the negative control. The plates were incubated at 37°C for 1 h, washed with PBST, and then incubated with HRP-anti-rabbit IgG (1:1000 dilution, 100 μl) at 37°C for 1 h. Finally, TMB (100 μl per well) was added and incubated at 37°C for 20 min, and 50 μl per well of 2 M H_2_SO_4 _was added to stop the enzymatic reaction. The plates were read with a ZS-2 microplate reader (Beijing Xin Feng Machine Electric Technical Instruments, Beijing, China). The final optical density reading was adjusted for background absorbance (OD = OD_450 nm _- OD_630 nm_).

We could observe an increase in the OD after each injection, indicating that the titers were rising. The pre-immune sera showed insignificant amount of absorbance, implicating that the antisera were specific. We determined that after four injections, a sufficient titer was achieved for both immunogens (OD reading of the sera of the fourth injection was at saturation at 1:200 dilution), and terminating bleeds were performed. The sera were collected as mentioned above and stored at -80°C.

### Competitive indirect ELISA

Two 96-well plates were each coated with 5 μg per well of CL or SM_2 _artificial antigen overnight at 4°C. The plates were washed 3 times with PBST, and blocked with 150 μl of 0.1% (w/v) OVA per well at 37°C for 1.5 h. A 1:12,800 dilution of the antisera were shown during tittering to achieve an OD in the working range, thus for both CL and SM_2_, 50 μl of a 1:12,800 dilution of the corresponding antiserum were applied to each well, and 50 μl of the corresponding hapten standard solutions was added as the "competitor". The concentrations of the standard solutions of CL were 0, 1, 5, 10, 20, 50, 100, 1000, 5000, 12,500, and 25,000 ng/ml; for SM_2_, the concentrations of the standard solutions were 0, 1, 10, 50, 100, 1000, 5000, 10,000 and 20,000 ng/ml. Each concentration was added to one row of twelve wells. The plates were incubated at 37°C for 1 h, and were processed the same way as in the indirect ELISA mentioned above.

### Protein microarray immunodetection

Twenty nanogram of each artificial antigen were spotted onto a 7 × 7 array on P-L-L microarray slides (CapitalBio Corporation, Beijing, China) using an OmniGrid-100 Microarrayer (Genomic Solutions, Ann Arbor, MI, USA). Following a 2 h incubation in a humid chamber at 37°C, the slides were inverted and immersed into PBS (pH 7.5) containing 0.2% OVA (w/v). The slides were then turned right side up and immersed in a 2% BSA solution for 1 h at room temperature with gentle agitation. Next, the slides were washed twice (10 s each) at room temperature with PBST and twice with ddH_2_O. The diluted antiserum (1:500 with PBS) and one of the corresponding standard hapten solutions (0, 5, 10, 20, 50, 100 and 1000 ng/ml) were mixed and added to an artificial antigen-spotted protein microarray slide. The slides were incubated with the antiserum-hapten mix for 1 h at room temperature and then washed three times with PBST and once with ddH_2_O. Then, cy3-goat-anti-rabbit IgG (1:4000 diluted in PBST) was applied to the slides. After 1 h of incubation, the slides were rinsed with PBST and then washed as previously. The slides were dried by centrifugation and scanned using an Axon GenePix^® ^4000B microarray scanner (Molecular Devices, Sunnyvale, CA, USA) to detect the fluorescence signal.

### IC_50 _determination

A dose-response curve was produced and was used to calculate the IC_50 _by non-linear regression analysis using GraphPad Prism 5 software (GraphPad Software, Inc, La Jolla, CA, USA) For the ci-ELISA, the final optical density reading was adjusted for background absorbance (OD = OD_450 nm _- OD_630 nm_). The log of the hapten concentrations were plotted against the percentage of inhibition, which was calculated by the equation (OD_sample_/OD_control_) × 100%, where the control group (i.e., 0 ng/ml hapten) OD was considered to be the point of 100% activity.

The results of the protein microarray assays were reported as the average pixels of fluorescence at 532 nm of the 49 spots for each hapten concentration minus background pixels. The IC_50 _of the protein microarray assays were determined by the same method of non-linear regression analysis as for the ci-ELISA.

### Fortification of muscle homogenate with clenbuterol

The muscle tissue samples in control group were used in the fortification studies. Fortification was carried out by adding 25 μl of methanolic solutions containing clenbuterol concentrations of 30, 120 and 300 ng/ml to the tissue homogenate, resulting in fortification levels of 0.5, 2 and 5 ng/g, respectively [12,13]. The fortified samples were used for calculation of recovery by ci-ELISA and protein microarrays.

### Preparation of muscle samples from clenbuterol-fed chicken

Fifteen eight-week-old broilers (AA species, Zhengda Co., Beijing, China) were randomly divided into five groups and raised in brooders provided with fresh feed and water every day. The control group was given drug-free feed; the other four groups were given feed treated with 3 mg/kg CL (w/w) [12-14]. The broilers were fed for 14 consecutive days. One treated group was slaughtered after 0, 1 day, 7 days, and 14 days withdrawal periods, respectively. The muscle tissue samples were collected and frozen at -20°C until analysis. The muscle tissue samples were homogenized using a DI 25 basic Ultraturrax homogenizer (Ika-Werke, Staufen, Germany). Five gram of homogenate was mixed with 25 ml 50 mM HCl by shaking for 1.5 h. The homogenate was centrifuged, and the supernatant was collected in a tube containing 300 μl of 1 M NaOH and mixed for 15 min. 400 μl of 0.5 M KH_2_PO_4 _were then added and the mixture was stored at 4°C overnight. The next day the mixture was centrifuged at 2750 g for 15 min, and the supernatant was purified by RP-18 cartridges (Supelco, Bellefonte, PA, USA). Clenbuterol was eluted with methanol from the purified supernatant as described elsewhere [15]. The eluent was collected by vacuum and the solvent was evaporated under a nitrogen stream. Dried residue was redissolved in 400 μl of water, and 20 μl per well were analyzed by ci-ELISA and per slide by protein microarray analysis. The concentrations of the recovered CL were calculated against a standard curve made with 0, 1, 5, 10, 25, 50, 100, 250, 500 and 1000 ng/ml CL solutions for both methods. The total CL recovered were converted to and reported as per gram of tissue.

## Results and Discussion

**Figure 1:**
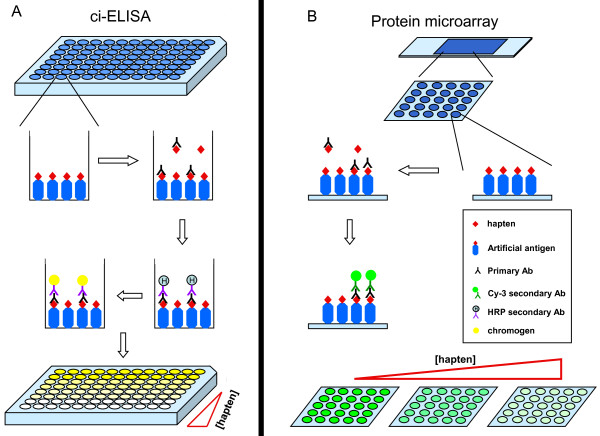
**Schematics of the ci-ELISA and protein microarray procedures**. 5 μg of the artificial antigens (CL or SM_2_) are coated onto the 96-well plates in (A) ci-ELISA, or 20 ng of the artificial antigens were spotted onto the protein microarray slides in (B) protein microarray. Antibodies against the particular artificial antigens are added together with a range of concentration of the corresponding hapten. As the haptens would compete with the immobilized artificial antigens for binding to the antisera, an increasing concentration of the hapten would result in a decreasing signal. For ci-ELISA, the bound antibodies are visualized by an anti-rabbit-HRP conjugated antibody with the addition of a chromogen (A). For the protein array, the secondary antibodies are conjugated to a fluorescent dye (Cy3), and the signal can be directly measured by a fluorescence microarray scanner (B).

**Figure 2:**
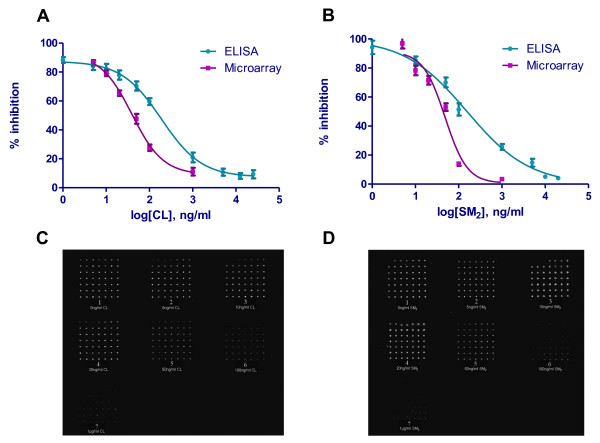
**IC_50 _of protein microarray immuntodetection and ci-ELISA**. Upper panels: Dose-response curves for (A) CL and (B) SM_2 _from ci-ELISA and protein microarray. The log of the hapten concentration (x-axis) was plotted against the percentage of inhibition (y-axis), which is (OD_sample_/OD_control_) × 100%. The control group OD was considered to be the point of 100% activity. The IC_50 _was determined by non-linear regression analysis. Lower panels: fluorescent signals from the protein microarray slides for (C) CL and (D) SM_2_. The concentration of the standard solution for each slide is: 1: 0 ng/ml; 2: 1 ng/ml; 3: 5 ng/ml; 4: 10 ng/ml; 5: 20 ng/ml; 6: 100 ng/ml; 7: 1000 ng/ml.

Protein microarray and competitive indirect ELISA (ci-ELISA) were carried out in parallel to compare their sensitivities for the detection of the haptens. Figure 1 shows the schematics of the two assays. The sensitivity was determined by measuring the IC_50 _of the haptens in competitively inhibiting the binding of the antisera to the immobilized artificial antigens. A dose-response curve was produced for each assay for the two haptens tested and was used to calculate the IC_50 _by non-linear regression analysis (Figure 2A and 2B). The classic sigmoidal fashion demonstrated the specificity of the antisera to the immobilized ligands. The IC_50 _of the haptens measured by ci-ELISA were 190.7 ng/ml for CL and 156.7 ng/ml for SM_2 _with a standard error of logIC_50 _of 0.06611 and 0.1169, respectively. On the other hand, The IC_50 _of the haptens measured by the protein microarray immunodetection were 39.6 ng/ml for CL and 48.8 ng/ml for SM_2 _with a standard error of logIC_50 _of 0.06247 and 0.06494, respectively. The scans of the microarray slides were shown in Figure 2C and 2D. The protein microarrays showed 4.5 and 3.5 times lower IC_50 _than the ci-ELISA detection for CL and SM_2 _respectively, suggesting that the protein microarray method has better sensitivity than the ci-ELISA detection method.

We next examined if the protein microarray assay would perform equally well with complex biological material. Chicken muscles fortified with known concentrations of CL were homogenized, and the eluted CL from each sample was being tested by the protein microarray and ci-ELISA. As shown in Table 1, both assays could recover effectively from the 5 ng/g CL-fortified tissues. However, when the fortifying CL concentration was reduced to 2 and 0.5 ng/g, the protein microarray could recover significantly more than ci-ELISA (~90% vs. ~76%). When we examined the recovery from CL-fed chicken muscle tissues, the protein microarray assay again showed higher sensitivity than ci-ELISA for samples with lower CL concentrations, i.e., longer withdrawal periods (7 days and 14 days) (Table 2).

**Table 1:** Recoveries of CL from fortified chicken muscle tissues by ci-ELISA and protein microarray

CL added (ng/g)	ci-ELISA		Microarray	
	
	CL detected (ng/g)	Recovery (%)	CL detected (ng/g)	Recovery (%)
0.5	0.38 ± 0.04	76 ± 7.8	0.45 ± 0.05	90* ± 9.9
2.0	1.54 ± 0.20	77 ± 9.9	1.84 ± 0.17	92* ± 8.5
5.0	4.25 ± 0.28	85 ± 5.6	4.50 ± 0.41	95 ± 8.6

Each experiment was repeated 5 times. *p < 0.05 by unpaired t-test.

% recovery = (CL detected/CL added) × 100%

**Table 2:** Detection of CL from CL-treated chicken muscle tissues by ci-ELISA and protein microarray

Withdrawal Time (day)	Detected CL ± SD (ng/g)	
	
	ci-ELISA	Microarray
0	45 ± 3	48 ± 2
1	4 ± 1.2	7 ± 1.5
7	1 ± 0.3	3 ± 0.5*
14	0.1 ± 0.04	0.9 ± 0.03**

Each experiment was repeated 3 times. *p < 0.05 and **p = 0.0001 by unpaired t-test.

The use of DNA microarrays [16] and immunobiosensor technology [17] in residue detection has become increasingly popular in recent years. However, the use of protein microarray technology for detecting toxic drug residue in food is relatively new. Our study established the use of protein microarray immunodetection of drug residues as a better method than a traditional method such as ci-ELISA. In our experiments, ci-ELISA underestimated the presence of CL in tissues when the concentration was below the ng/g range. The protein microarray displayed much more consistent recovery and higher sensitivity. The fluorescence nature of the readout method of the protein microarray is certainly more sensitive than the colorimetric measurement in ci-ELISA. Moreover, the measurement by protein microarray is more accurate and reliable, as each sample can be assayed in much higher number of repeats (49 spots on the microarray vs. 12 wells in ci-ELISA). The protein microarray method also consumed far less samples than ci-ELISA. Thus, the protein microarrays have the added advantages of requiring fewer reagents, a faster analysis and the potential to be a multi-analyte platform.

## Conclusions

Our study has shown that protein microarray technology is a more sensitive, reliable and efficient method for small molecule detection than traditional ELISA.

## Abbreviations

BSA: bovine serum albumin; CL: clenbuterol; SM_2_: sulfamethazine; OVA: ovalbumin; ci-ELISA: competitive indirect-ELISA; HPLC: high performance liquid chromatography.

## Competing interests

The authors declare that they have no competing interests.

## Authors' contributions

LZ involved in the experimental design, data interpretation and manuscript revision. WZ carried out most of the experiments. CZ made contributions to samples and manuscript preparation. KG involved in data collection and manuscript preparation. XG made contributions to data analysis and statistical modeling. KHK involved in data analysis and manuscript revision. All authors read and approved the final manuscript.
